# Ribonuclease from* Bacillus* Acts as an Antiviral Agent against Negative- and Positive-Sense Single Stranded Human Respiratory RNA Viruses

**DOI:** 10.1155/2017/5279065

**Published:** 2017-05-04

**Authors:** Raihan Shah Mahmud, Christin Müller, Yulia Romanova, Ahmed Mostafa, Vera Ulyanova, Stephan Pleschka, Olga Ilinskaya

**Affiliations:** ^1^Institute of Fundamental Medicine and Biology, Kazan Federal University, Kremlevskaya Street 18, Kazan 420008, Russia; ^2^Institute of Medical Virology, Justus Liebig University, Schubert Street 81, 35392 Giessen, Germany; ^3^Center of Scientific Excellence for Influenza Viruses, National Research Centre (NRC), El-Buhouth Street 87, Dokki, Cairo 12311, Egypt

## Abstract

*Bacillus pumilus* ribonuclease (binase) was shown to be a promising antiviral agent in animal models and cell cultures. However, the mode of its antiviral action remains unknown. To assess the binase effect on intracellular viral RNA we have selected single stranded negative- and positive-sense RNA viruses, influenza virus, and rhinovirus, respectively, which annually cause respiratory illnesses and are characterized by high contagious nature, mutation rate, and antigen variability. We have shown that binase exerts an antiviral effect on both viruses at the same concentration, which does not alter the spectrum of A549 cellular proteins and expression of housekeeping genes. The titers of influenza A (H1N1pdm) virus and human rhinovirus serotype 1A were reduced by 40% and 65%, respectively. A preincubation of influenza virus with binase before infection significantly reduced viral titer after single-cycle replication of the virus. Using influenza A virus mini genome system we showed that binase reduced GFP reporter signaling indicating a binase action on the expression of viral mRNA. Binase reduced the level of H1N1pdm viral NP mRNA accumulation in A549 cells by 20%. Since the viral mRNA is a possible target for binase this agent could be potentially applied in the antiviral therapy against both negative- and positive-sense RNA viruses.

## 1. Introduction

The single stranded RNA viruses, such as negative-sense Ebola, Marburg, Lassa, and influenza and positive-sense human immunodeficiency viruses, are very important human pathogens in the world. Recent virus outbreaks with a large number of human deaths were caused by RNA viruses like Ebola, corona, Zika, and different strains of influenza A viruses. Among RNA viruses, the respiratory viruses are highly contagious and cause annually worldwide epidemics and occasional pandemic outbreaks. The influenza A virus even without a pandemic outbreak kills up to half million humans each year. A great number of diseases are attributable to human rhinoviruses (HRV) which are the major cause of the common cold. HRV infections are suffered by everyone. Recent reports suggest that HRV infection is associated with severe respiratory illness in children [1].

The main problem of antiviral therapy is drug resistance. Although the majority of common viral diseases are self-limited illnesses that do not require specific antiviral therapy, antiviral drugs help to prevent complications or shorten the severity and duration of viral diseases symptoms. Considerable economic losses from annual epidemics promote constant search for new antiviral agents, which become useless with time due to the high variability of viruses.

Usually, two groups of drugs are used for the treatment of influenza. The first group including rimantadine and its analogs inhibits the ion channel function of M2 protein, thereby inhibiting viral uncoating. The second one represented by oseltamivir inhibits the enzyme neuraminidase [3]. Inducing adaptive immunity through the vaccination with inactivated or attenuated antigenic material of viruses helps to prevent influenza illness or to reduce its impact in individuals. There is no permanent vaccination against influenza virus and each year countries lose a large amount of money from their budget to prepare new vaccines and rescue the national public health and life. Widespread antiviral resistance due to the high mutation rate in viral genome limits the clinical impact of all types of medicinal products for the prevention and treatment of influenza infections.

Currently, there is no vaccine against HRV [1]. The HRV has almost the highest number of serotypes (more than 100) among all respiratory viruses due to mutations of its genome. Antigenic heterogeneity of HRVs is regarded as a major barrier to effective vaccine development which has to be highly polyvalent and has resulted in little progress over 50 years [1, 4]. Other problems, which have impeded rhinoviral vaccine development, include the lack of convenient animal models and as expected the absence of great impact on respiratory morbidity and mortality while reducing the frequency of common colds [5]

Today, there are no drugs that destroy viruses; all antiviral agents can only inhibit viral development. Therefore, degradation of the viral RNA seems to be a very promising approach of antiviral therapy. The earliest studies on the antiviral activity of RNases were performed using pancreatic RNase as an agent that quickly normalized the state and decreased the symptoms of meningitis and cerebrospinal pleocytosis in patients with tick borne encephalitis [6]. Shortcoming of mammalian RNases is their affinity to cytosolic RNase inhibitor protein (RI) in human cells by which adventitious mammalian RNases are inhibited. In contrast, bacterial RNases are not inhibited by RI and can retain their catalytic activity in mammalian tissues. One of the well-studied bacterial RNases is binase, the guanyl-preferring low molecular weight RNase secreted by* Bacillus pumilus* [7].

We proposed earlier that mechanisms of antiviral activity of RNases include both the direct action on nucleic acid and indirect effects, that is, intervention into the RNA interference, immunomodulation, and induction of infected cell apoptosis (for review see [8]). We have demonstrated that binase reduced the titer of pandemic influenza A/Hamburg/4/2009 (H1N1pdm), reovirus serotype 1 (Reo 1-Lang), herpes virus type I (pseudorabies), Middle East respiratory syndrome coronavirus (MERS-CoV), and human corona virus (HCoV-229E) in infected Madin-Darby canine kidney (MDCK) epithelial cells, African green monkey kidney (Vero) cells, Madin-Darby bovine kidney (MDBK) epithelial cells, human fetal lung fibroblast (MRC5) cells, and hepatocellular carcinoma (Huh7) cells, respectively [9–11]. Influenza viruses contain single stranded negative-sense RNA; reoviruses have a double stranded RNA genome, while herpes virus possesses genomic DNA. Because of the specific catalytic activity of binase towards RNA (mainly single stranded) and its inhibitory effect towards RNA- and DNA-containing viruses, it could be proposed that this enzyme should affect mRNA, which is synthesized by all viruses. The aim of our study was to prove that viral mRNA is a direct target of binase.

## 2. Materials and Methods

### 2.1. Cells, Viruses, and Stocks

A546 (human lung adenocarcinoma epithelial), HeLa (human cervical cancer), and MDCK-II (Madin-Darby canine kidney epithelial) cells were cultured in growth Dulbecco's modified Eagle medium (DMEM) (Gibco, USA) supplemented with 10% fetal bovine serum (PAA, Austria), 100 U/ml penicillin, and 0.1 mg/ml streptomycin (P/S) (Gibco, USA) and incubated at 37°C in a 5% CO_2_ atmosphere. The pandemic influenza A/Hamburg/04/09 (H1N1pdm) and human rhinovirus serotype 1A (HRV1A) were provided by the strain collection of the Institute of Medical Virology, Justus Liebig University, Giessen, Germany, and propagated in MDCK-II and HeLa cells, respectively. Briefly, MDCK-II and HeLa monolayers were incubated with H1N1pdm and HRV1A at a multiplicity of infection (MOI) of 1 diluted in PBS^++^/BA (PBS containing 0.2% bovine albumin (PAA, Germany), 1 mM MgCl_2_, 0.9 mM CaCl_2_, 100 U/ml penicillin, and 0.1 mg/ml streptomycin). After 1 h of virus adsorption in the dark, the cell monolayers were supplemented with medium and incubated at either 37°C (H1N1pdm) or 33°C (HRV1A) in humidified 5% CO_2_ conditions.

### 2.2. Bacteria Growth Condition and Binase Isolation

Binase (EC 3.1.27.3; bacterial extracellular guanyl-preferring ribonuclease) was collected from the culture medium of* Bacillus pumilus* B3073 (the former name is* B. intermedius* B3073 [7]). The cultures were grown in a 1 L conical flask containing 1/5 flask volume of the phosphate deficient liquid medium (2.0% low phosphate peptone, 1.0% glucose, 1.0% CaCl_2_, 0.3% NaCl, 0.03% MgSO_4_  × 7H_2_O, and 0.01% MnSO_4_, pH 8.5) with 200 rpm in a Multitron shaking incubator (INFORS HT, Switzerland) at 37°C until the beginning of the stationary phase of bacterial growth. After centrifugation at 6000*g* for 30 min, the pelleted bacterial cells were removed; the supernatant was acidified by glacial acetic acid to pH 5.0, diluted 1 : 5 in double-distilled water (ddH_2_O), and applied on the column with DE-32 cellulose (Whatman, UK) equilibrated with 10 mM sodium acetate buffer (pH 5.0). The flow through from DE-32 was transferred to column packed with phosphocellulose P11 (Whatman, UK) equilibrated with 10 mM sodium acetate buffer (pH 5.0). The system was then equilibrated with 20 mM sodium phosphate buffer (pH 7.0). Binase was eluted with 200 mM sodium phosphate buffer (pH 7.0). Further polishing purification of binase was performed using the UnoS column and BioLogic DuoFlow chromatography system (Bio-Rad, USA) equilibrated with 20 mM sodium acetate buffer (pH 5.0) and eluted using a linear gradient of 0–0.25 M NaCl. Elution fractions of 5 ml were collected. The protein samples were frozen overnight at −20°C, followed by freezing for 15 min at −80°C and then lyophilized for 48 h at −50°C under vacuum using a Labconco FreeZone 2.5 Liter Freeze Dry System (Labconco, USA). Homogeneity and authenticity of the purified enzyme were confirmed by PAAG electrophoresis, Western blotting, and MALDI TOF/TOF mass spectrometry on UltrafleXtreme (Bruker Corporation, Germany) as described in [12].

### 2.3. Enzymatic Activity of Binase

The ribonuclease activity of binase was registered by 260 nm absorbance measuring of the products of binase hydrolysis of high molecular weight yeast RNA. Binase was incubated in 0.25 M Tris-HCl buffer (pH 8.5) in the presence of 0.5 *μ*g/*μ*l RNA (final concentration) in a water bath for 15 min at 37°C. The reaction was stopped by addition of ice cold 6.8% perchloric acid followed by incubation on ice bath for 10 min followed by centrifugation for 10 min at 12000*g*. The supernatant was diluted 1 : 20 in ddH_2_O and used for the 260 nm absorbance measurement using spectrophotometer SmartSpec Plus (Bio-Rad, USA). One unit of ribonuclease activity corresponded to the quantity of enzyme that increased the extinction of acid-soluble products of RNA hydrolysis at 260 nm by 1 optical unit after incubation for 1 h at 37°C, calculated per ml of the enzyme solution.

### 2.4. Ribonuclease Activity in Cell Culture Medium

To identify the enzymatic activity of binase, the growth medium from subconfluence (90–95%) A549 cells in a 96-well plate (in triplicate) was replaced with 100 *μ*g/ml binase (final concentration) containing complete DMEM and incubated for 48 h at 37°C in 5% CO_2_. The supernatant (cell culture fluid) containing binase was collected to measure the binase activity using RNase activity assay as described before (see Section 2.3).

### 2.5. Cell Viability Measurement

The cytotoxic concentration (CC_50_, the concentration of compound that causes death to 50% of viable cells) of HeLa cells under binase treatment was measured using MTT assay as described by Shah Mahmud and Ilinskaya [13]. Briefly, growth medium of subconfluence HeLa cells in a 96-well plate (in triplicate) was replaced with DMEM containing different concentration of binase (at final concentrations of 0.005–500 *μ*g/ml) and kept for 24 h at 33°C in a humidified 5% CO_2_ incubator. After aspirating the media and washing with PBS^++^ (phosphate-buffered saline containing 1 mM MgCl_2_, 0.9 mM CaCl_2_) the cells were left to recover for 60 min in growth medium and then replaced by 200 *μ*l of MTT-mix (growth DMEM medium containing 0.123 mg/ml of MTT-reagent = 1-(4,5-dimethylthiazol-2- yl)-3,5-diphenylformazan, Sigma Aldrich, USA). The cells were further incubated for 60 min at 37°C in 5% CO_2_. After discarding the MTT-mix, the cells were fixed with 4% PFA paraformaldehyde (Roth, Germany) for 30 min at RT (room temperature) and then the fixing solution was discarded. The cells were air dried for 10–15 min and the tetrazolium crystals were dissolved by adding 200 *μ*l of isopropanol to each well. The plates were shaken for 10 min and analyzed photometrically at 492 nm excitation in an ELISA reader EL808 (BioTek, USA). The MTT value of the binase nontreated control was used as 100%. The percentage of cell viability after binase exposure was calculated as follows: percentage viability = 100/(MTT value of control × MTT value of binase-treated sample). The CC_50_ was determined using GraphPad Prism 5.0 Software (GraphPad Software Inc., USA) by plotting the percentage of viable cells as a function of the compound concentration.

### 2.6. Binase Treatment

To determine the effect of 100 *μ*g/ml binase against H1N1pdm virus, the A549 monolayers were subcultured to prepare 96-well plates (10^6^ cells/well, *n* = 8). Following the overnight 90–95% confluency of the monolayers, the growth medium was removed and cell monolayers were washed with PBS^++^. Virus suspension containing H1N1pdm with MOI = 1 diluted in PBS^++^/BA (50 *μ*l) was added to each well and incubated for 1 h at RT. The viral supernatant was then removed and binase at a final concentration of 10 or 100 *μ*g/ml in an infection medium (DMEM containing 100 U ml^−1^ penicillin, 100 *μ*g/ml streptomycin, 0.2% BSA (PAA, Germany), and 1 *μ*g/ml TPCK-treated trypsin) was added. The cells were incubated for 12 h and then the viral loads in the collected supernatants were estimated using focus assay as described in [13, 14]. The cell monolayers were subjected to cellular total protein or RNA extractions for further investigations. The binase and virus preincubation for 45 min and infection and incubation steps were performed as previously described by Shah Mahmud and Ilinskaya [13].

### 2.7. Binase Effective Concentration Determination

The effective concentration (EC_50_, the concentration of compound which reduces the virus titer by 50%) of binase against HRV1A was determined in HeLa cells. The cells were grown in a 48-well plate (in triplicate) overnight at 37°C and 5% CO_2_. Cell monolayers were then washed and infected with 75 *μ*l of virus suspension in PBS^++^/BA (MOI = 1) for 1 h at 33°C. After removing the virus inoculum, cells were incubated with 250 *μ*l growth medium in each well containing different concentrations of binase (at final concentrations of 0.01–500 *μ*g/ml) for 24 h, at 33°C and 5% CO_2_. The supernatants containing HRV1A virus were assayed for their viral titers using plaque assay.

### 2.8. Virus Titration

The HRV1A titers were determined in a 24-well cell culture plate containing subconfluence (90–95%) HeLa cell monolayers using plaque assay. Briefly, the HRV1A virus inoculum was diluted 1 : 10 in PBS^++^/BA. The growth media from HeLa cells were removed and the cell monolayers were washed using PBS^++^. The diluted virus was added to each well and incubated for 1 h at 33°C and 5% CO_2_ and then removed. 500 *μ*l of Avicel-media containing 1x MEM and 1.25% Avicel (FMC BioPolymer, Belgium) was added and cells were further incubated for 48 h at 33°C with 5% CO_2_. After removal of the overlay media, the cell monolayers were washed with PBS^++^, stained with 0.1% crystal violet solution, further washed, and dried. The plaques corresponding to propagating viral particles were counted. The H1N1pdm titers were determined in MDCK-II using focus assay. To analyze the binase effect against H1N1pdm or HRV1A and identify the EC_50_ of binase, the plaques were counted and PFU/ml was measured using MS Excel Software package. The PFU/ml values of the binase nontreated infected HeLa cells were used as 100%. The EC_50_ was determined using GraphPad Prism 5.0 Software (GraphPad Software Inc., USA) by plotting the percentage of viable cells as a function of the compound concentration.

### 2.9. Protein Extraction from Cells

To perform a two-dimensional (2D) difference gel electrophoresis (DIGE) protein expression analysis, the total cellular proteins of binase-treated A549 cells (100 *μ*g/ml) were extracted after 12 h of incubation in 6-well plates (in triplicate). Binase nontreated cells were taken as control. After removal of the cell culture media, the cell monolayers were washed twice with PBS^++^, scratched off, and collected in 1 ml PBS. The cells were then pelleted by centrifugation for 30 sec at 13000*g* and 4°C. The supernatant was discarded and cell pellets were lysed on ice using lysis buffer (TLB: 25 mM Tris, pH 8.0; 137 mM NaCl; 10% glycerol; 0.1% sodium dodecyl sulphate; 0.5% sodium deoxycholate; 1% NP-40; 2 mM EDTA, pH 8.0; 0.2 mM Pefablock; 5 *μ*g/ml aprotinin; 5 *μ*g/ml leupeptin; 1 mM Na-vanadate; and 5 mM benzamidine). After adding the lysis buffer, the cells were incubated for 10 min on ice and successively centrifuged for 30 min at 13000*g* with 4°C and supernatants were collected. The protein concentration of each sample was determined according to the manufacturer's instruction using Bradford Assay Kit (Bio-Rad, USA).

### 2.10. Cyanine Dye Labeling of Protein Samples

Total cellular proteins (100 *μ*g) from binase nontreated (control) and binase-treated samples were labeled with green (Cy3) and red (Cy5) cyanine dyes at a concentration of 800 pmol dye/100 *μ*l of proteins, respectively. The dye and protein mix were incubated on ice for 1 h in the dark at RT. The labeling reaction was terminated by incubating with 10 mM lysine for 10 min. The labeled protein was mixed and used for isoelectric focusing (IEF).

### 2.11. 2D Difference Gel Electrophoresis

The isoelectric pH gradient (IPG) tube gel was prepared using 8 M urea; 4% acrylamide; 2.4% ampholytes; 2.4% CHAPS; and NP40 according to Protean IEF Cell (Bio-Rad, USA) manufacturer's instructions. 100 *μ*g of each labeled protein mix from control and test samples was combined in a 50 *μ*l standard buffer (8 M urea; 4% CHAPS and NP-40; 10 mM Tris-HCl, pH 8.5) containing 2% DTT (dithiothreitol) and added onto the prepared IEF gel in a tube. The first-dimensional isoelectric focusing was carried out on a PROTEAN II xi Cell (Bio-Rad, USA) chamber at RT using the following program: 100 V for 1 h, 200 V for 1 h, a gradient of 200 V–700 V for 6 h, 700 V for 5 h, 900 V for 1.5 h, and 1000 V for 1 h.

The 9–16% polyacrylamide gradient gel for two-dimensional gel electrophoresis was prepared according to Protean IEF Cell (Bio-Rad, USA) manufacturer protocol. The IEF gel was equilibrated for 15 min in SDS-PAAG equilibration buffer (6 M urea; 0.375 M Tris-HCl, pH 8.8; 2% SDS; 20% glycerol; 2% (w/v) DTT) for 15 min. The IEF gel was carefully placed on top of 9–16% SDS-PAAG with 400 ml upper electrolyte (20 mM NaOH) on glass plate. 2 liters of lower electrolyte contained 20 mM of H_3_PO_4_. The electrophoresis was carried out using the following program: 20 mA for 20 min, 40 mA for 2 h, and 35 mA for 3 h. The proteins separated on gels covering the 15–150 kDa range. A Typhoon FLA 9500 Imager (GE Healthcare, UK) was used to visualize protein spot on the gels. The excitation and emission wavelength combinations of 532 nm and 635 nm were used to capture the images of protein labeled with Cy3 and Cy5, respectively. The spot detection matching was carried out by using ImageQuant TL software (GE Healthcare, UK).

### 2.12. Plasmids and Primers

For the in vitro reconstitution of viral ribonucleoprotein (vRNP) complex, four plasmids pHW-PB1-Hamburg, pHW-PB2-Hamburg, pHW-PA-Hamburg, and pHW-NP-Hamburg generating the mRNAs of polymerases and NP segments of H1N1pdm virus together with pPol1-GFP-RT generating a vRNA-like Pol1-transcript encoding the green fluorescent protein (GFP) were used. GFP, a reporter protein in negative-sense, are flanked by the 3′- and 5′-noncoding region of the NS RNA segment of influenza A/WSN/33 virus [2] placed between a truncated human RNA polymerase I promotor (POLI) and hepatitis delta virus ribozyme (R). The expressed subunits of the viral polymerases and nucleoprotein replicated and transcribed the influenza virus-like RNA expressed by pPOLI-GFP-RT into mRNA.

To perform the real-time RT-PCR the primer pairs (Actin_SYBR_F: 5′-AGG CAC CAG GGC GTG AT-3′; Actin_SYBR_R: 5′-GCC CAC ATA GGA ATC CTT CTG AC-3′ and GAPDH_SYBR_F: 5′-CCA GGT GGT CTC CTC TGA CTT C-3′; GAPDH_SYBR_R: 5′-CAA AGT GGT CGT TGA GGG CAA TG-3′) were used to amplify the cellular ACTB (beta-actin) and GAPDH (glyceraldehyde-3-phosphate dehydrogenase) genes, respectively. The H1N1pdm viral NP mRNA specific primer pairs (HH_SYBR_F: 5′-GGC CAT AAG GAC CAG GAG TG-3′; HH_SYBR_R: 5′-CCG CTG AAT GCT GCC ATA AC-3′) were used to identify the binase effect on influenza A viral RNA using real-time RT-PCR.

### 2.13. RNA Extraction and cDNA Preparation

To identify the binase action on the cellular and viral mRNA expression levels in A549-infected cells with H1N1pdm, binase-treated/infected, binase-treated/noninfected, noninfected, and nontreated A549 cells were kept for 12 h in 6-well plate (in triplicate). After washing with PBS^++^, the scratched cells were collected in PBS and immediately counted. 10^6^ cells from each sample were used for RNA extraction and cDNA preparation. The cells from each sample were lysed with RLT buffer (Qiagen, Germany). Total RNA was purified using a RNeasy mini Kit (Qiagen, Germany) according to recommended protocol. The total RNAs of each sample were eluted from each filter using the same volume of elution buffer. Equal volumes of total RNA were used to amplify A549 cellular mRNA of ACTB, mRNA of GAPDH, and H1N1pdm viral NP mRNA genes cDNA using MMLV RT Kit (Evrogen, Russia). In brief, 0.5–2 *μ*g of each total RNA in equal volume of each eluted sample was mixed with 20 *μ*M specific reverse primer, preincubated for 2 min at 70°C, and then immediately snap-cooled on ice. The reverse transcription was performed in 20 *μ*l solution containing preprepared RNA and primer mix, 100 U of MMLV reverse transcriptase, 1 mM of dNTP mix, 2 mM of DTT, 1x buffer of MMLV RT Kit (Evrogen, Russia), and 1 U RiboLock RNase Inhibitor (Thermo Fisher Scientific, USA). The RT-PCR was performed for 60 min at 42°C. The cDNA of each sample were diluted 1 : 10 using RNase free water (Thermo Fisher Scientific, USA) and used as a template for real-time PCR.

### 2.14. Real-Time PCR

To quantify the cellular and viral mRNA in A549 cells using qPCRmix-HS SYBR Kit (Evrogen, Russia), the 4 *μ*l of each cDNA (1–100 ng to each reaction) (see Section 2.13), 0.4 *μ*M of each (forward and reverse) primers, and 1x qPCRmix-HS SYBR (Evrogen, Russia) were mixed in a LightCycler 480 Multiwell Plate 96 (Roche Diagnostics, Switzerland) on ice in the dark and covered by LightCycler 480 Sealing Foil. Thermal cycling was done on a LightCycler 480 (Roche Diagnostics, Switzerland) system under the following conditions: 10 min at 95°C; 40 cycles of 15 sec at 95°C; 30 sec at 60°C; and 30 sec at 30°C. The quantification and data analysis was performed using the LightCycler 480 Service Software (Roche Diagnostics, Switzerland). For identification of binase action on cellular mRNA or H1N1pdm viral NP mRNA accumulation according to the relative fold-difference of expression levels (relative changes in gene expression from real-time quantitative PCR experiments) using internal control, mRNA expression data of a reference GAPDH gene was used to correct the control/calibrator (binase nontreated) and test/experimental (binase-treated) samples data. The ΔCt value of the calibrator from influenza virus-infected and binase nontreated cells (Ct_target_ (Ct value obtained using H1N1pdm viral NP mRNA specific primers in A549 cells) −Ct_reference_ (Ct value obtained using GAPDH mRNA primers in A549 cells)) and ΔCt value of the test from influenza virus-infected and binase-treated cells (Ct_target_ − Ct_reference_) were calculated. The fold change was calculated using 2^−ΔΔCt^ value, where ΔΔCt = ΔCt, test − ΔCt, calibrator.

### 2.15. Transfection

To investigate the effect of binase on the gene expression activity of H1N1pdm virus, 1 *μ*g of every five plasmids (see Section 2.12) encoding viral ribonucleoprotein complex together with pPOLI-GFP-RT was cotransfected into A549 cells as previously described with minor modifications (in triplicate) [15, 16]. Briefly, the transfection mixture consisting of 180 *μ*l “Opti-MEM” (Thermo Fisher Scientific, USA) and total of 5 *μ*g of plasmid DNA along with 12 *μ*l “TransIT-2020” (Mirus Bio, USA) was incubated for 45 min at RT. Then, the mixture was diluted to 1 ml using “Opti-MEM” and transferred to 80–90% confluent A549 cell monolayer (grown in 6-well plate) to allow transfection. The cells were then kept for 12 h at 37°C in 5% CO_2_ incubator. The transfection medium was replaced with 2 ml of Opti-MEM containing 0.2% BA and P/S with binase (10 *μ*g/ml) or without binase. After 36 h, the GFP expression was visualized and counted by Axiovert 35 inverted fluorescent microscope (Zeiss, Germany).

### 2.16. Statistical Analysis

Statistical tests and graphical data presentation were performed using GraphPad Prism 5 Software (GraphPad Software, Inc., USA) and MS Excel 2010 (Microsoft, USA). All data are presented as the mean ± standard deviation of the mean (SD). The significance between two groups was determined via “Student* t*-test.” A significant difference was considered to be a *P* value of <0.05.

### 2.17. Biosafety

All experiments involving influenza A (H1N1pdm) and rhinovirus serotype 1A (HRV1A) were performed using biosafety level 2 containment laboratory approved for such use by the local authorities (RP, Giessen, Germany).

## 3. Results and Discussion

**Figure 1:**
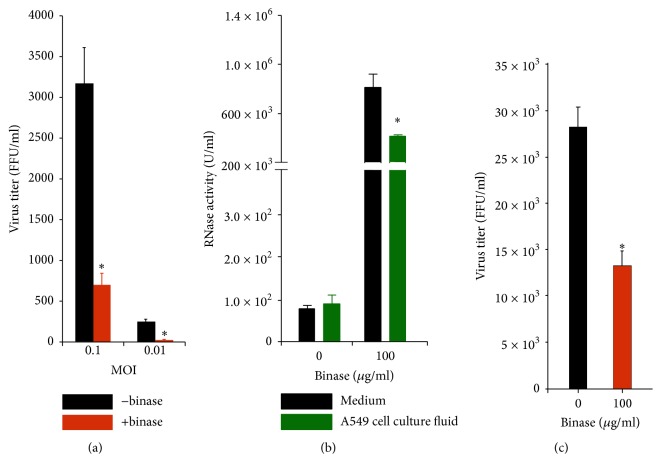
(a) Decrease of H1N1pdm virus titer in A549 cells infected by virus at an MOI of 0.01 and 0.1 which was preincubated with 10 *μ*g/ml binase for 45 min prior to cell infection; (b) binase catalytic activity in cell-free medium and A549 cell culture medium after 48 h incubation; (c) virus titer after 12 h incubation of A549 cells treated by 100 *μ*g/ml binase and infected by H1N1pdm virus at an MOI of 1. FFU/ml were calculated using supernatant after single-cycle replication of virus. Statistical significance was calculated using GraphPad Prism 5.0 Software and assessed by Student's* t*-test: ^*∗*^*P* < 0.001.

In the present work, we have studied the effect of bacterial ribonuclease binase on infectious titer of negative-sense single stranded RNA influenza A (H1N1pdm) virus after its single-cycle replication in A549 cell culture (12 h p.i.). In contradistinction to the previous report on the maximum anti-influenza effect of binase after multicycle virus replication [13], here, we have focused on the time period of 12 h p.i. to study virus gene expression at the moment of first round progeny virions formation. It was found that 45 min preincubation of binase at concentration of 10 *μ*g/ml with virus led to decrease in infectious virus titer by 5-fold at an MOI of 0.1 (Figure 1(a)). At an MOI of 0.01 the reduction of virus titer after binase treatment was more significant (8-fold). The higher antiviral effectiveness of binase on lower virus titer suggests the possible administration of binase at the early phase of virus infection or for prophylaxis.

The catalytic activity of binase (100 *μ*g/ml) in A549 cell culture media decreased to 50% after 48 h incubation (Figure 1(b)) that corresponded to the data obtained earlier for 1 *μ*g/ml binase [13]. Binase is a thermo- and pH-stable agent, which can be used in vivo retaining its activity. The 49% reduction of binase activity in the cell culture fluid can be explained by internalization of the enzyme to the A549 cells. Binase internalization to A549 cell cytoplasm and nucleus was previously illustrated by immunofluorescence methods [17]. Possible inhibition of catalytic activity and partial degradation of binase by extracellular proteases are not excluded though. Thus, binase acts as the antiviral agent under wide range of concentrations after short and long period of incubation. Moreover, the additional binase administration is not needed during 48 h.

**Figure 2:**
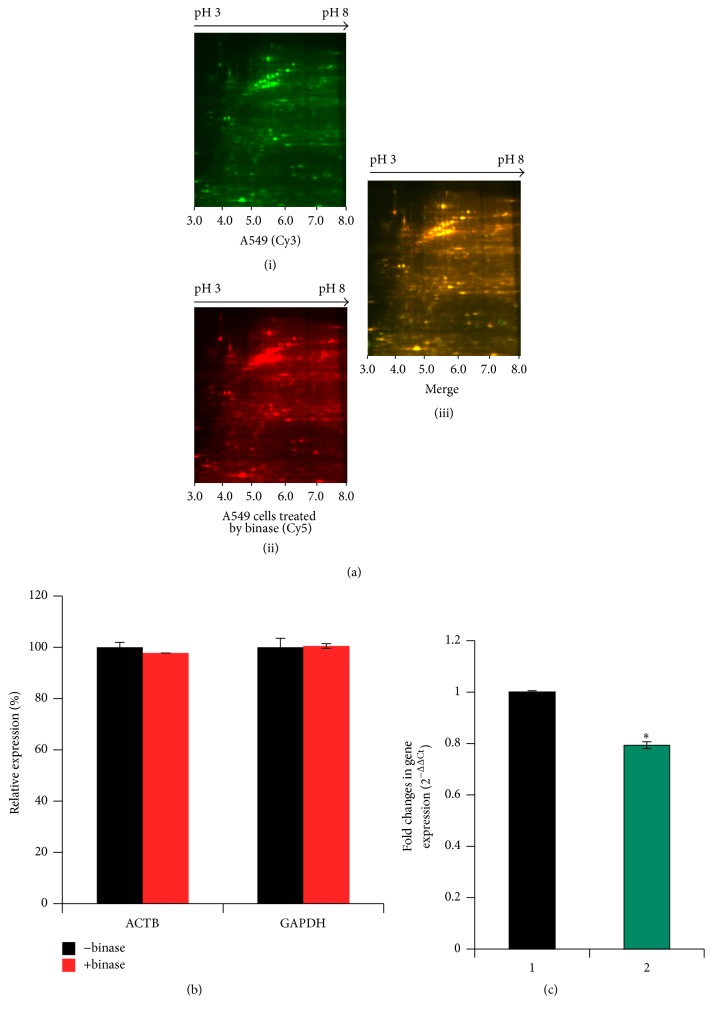
(a) Influence of 100 *μ*g/ml binase at 12 h p.i. on proteome of A549 cells. Two-dimensional difference gel electrophoresis (2D-DIGE) of proteins isolated from nontreated ((i), green labeling by Cy3) and binase-treated cells ((ii), red labeling by Cy5); (iii) merged image, unchanged protein spots appeared yellow. High resolution 2D-DIGE images were generated using Typhoon FLA 9500 Imager; samples were separated using narrow range of IEF tube gel, pH 3–8; gels cover 15–150 kDa range. (b) Real-time RT-PCR analysis of mRNA transcripts of housekeeping genes (beta-actin, ACTB; glyceraldehyde-3-phosphate dehydrogenase, GAPDH) in noninfected, binase-treated (100 *μ*g/ml, 12 h), and nontreated A549 cells. (c) H1N1pdm viral NP mRNA in infected nontreated A549 cells (1) and treated by 100 *μ*g/ml binase after 12 h (2). GAPDH mRNA was used as a loading control to normalize viral mRNA accumulation. 10^6^ cells from each sample were used to measure mRNA levels using Roche Light Cycler 480 system and target-specific primers. Statistical significance was calculated using GraphPad Prism 5.0 Software and assessed by Student's* t*-test: ^*∗*^*P* < 0.01.

It is known that binase at concentrations up to 100 *μ*g/ml is not toxic towards A549 cells [13, 17] while inhibiting H1N1pdm virus propagation [8, 13]. We have shown here that a concentration of binase of 100 *μ*g/ml without preincubation of the virus (at an MOI of 1) with binase reduced the infectious titer in A549 cell culture by 40% after a single replication round (Figure 1(c)). Therefore, binase seems to be able to act intracellularly on viruses before they exit the cells. This raises a question regarding antiviral targets of binase in the virus-infected cells. We performed a 2D-DIGE of binase-treated and nontreated cells. It was determined that binase at 100 *μ*g/ml does not alter the spectrum of A549 cellular proteins (Figure 2(a)). Moreover, the expression of cellular housekeeping genes, measured by mRNA levels of genes encoding structural protein ACTB and metabolic protein GAPDH, was not changed upon treatment of A549 cells with 100 *μ*g/ml of binase followed by 12 h incubation (Figure 2(b)). At the same time, binase reduced H1N1pdm viral NP mRNA accumulation in the A549 cells infected with virus at an MOI of 1 (Figure 2(c)), probably, reflecting the specific reduction of viral RNA. Expression of the gene was 20% lower in binase-treated virus-infected cells than in the virus-infected binase nontreated cells. These results support the idea of an inhibitory effect of binase on virus replication (Figure 1(c)).

**Figure 3:**
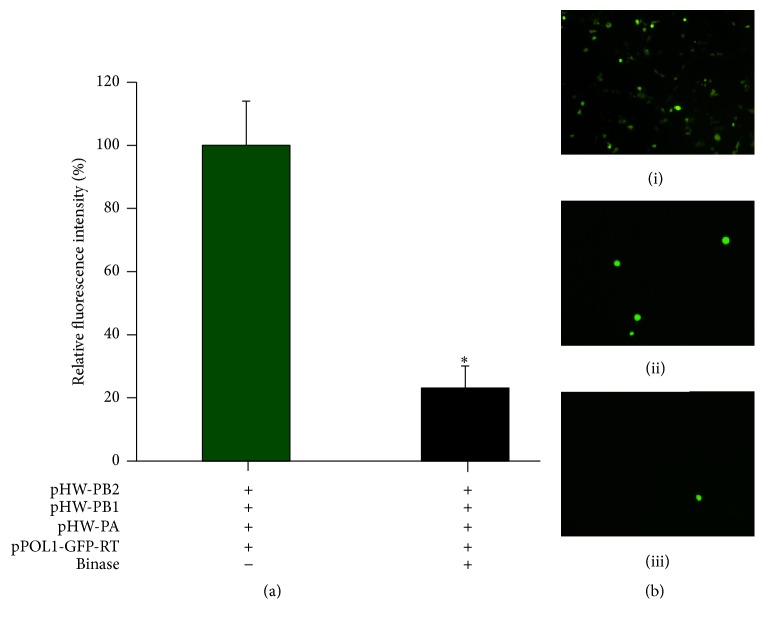
Effect of binase on functional H1N1pdm viral RNP polymerase complex formation in A549 cells transfected by plasmids (pHW) encoding vRNP subunits (PB1, PB2, and PA) and NP of H1N1pdm virus together with vRNA-like transcript pPOLI-GFP-RT encoding GFP. Decrease of GFP expression in the cells treated by binase (a) and visualization of this effect using Axiovert 35 inverted fluorescent microscope (b). (i) A549 cells carrying PAM 503 plasmid as GFP expression control; (ii) A549 cells without binase treatment; (iii) A549 cells treated by 10 *μ*g/ml binase after transfections. Statistical significance was calculated using GraphPad Prism 5.0 Software and assessed by Student's* t*-test: ^*∗*^*P* < 0.001.

To gain a more detailed evidence of binase effect on H1N1pdm viral gene expression in A549 cells we used a plasmid based reverse genetic system containing a set of plasmids encoding PB1, PB2, PA, and NP genes of H1N1pdm viral RNP complex and an influenza virus-like RNA encoding a GFP reporter protein (see Sections 2.12 and 2.15). GFP fluorescence is detectable only if plasmid-encoded genes for the RNP complex and the virus-like RNA are expressed. It was determined that binase reduced the viral RNA expression by 4.4 times indicating the binase inhibition of the expression of viral genes (Figures 3(a) and 3(b)). Therefore, a target of binase antiviral action could be viral mRNA. Its degradation prevents synthesis of viral proteins.

**Figure 4:**
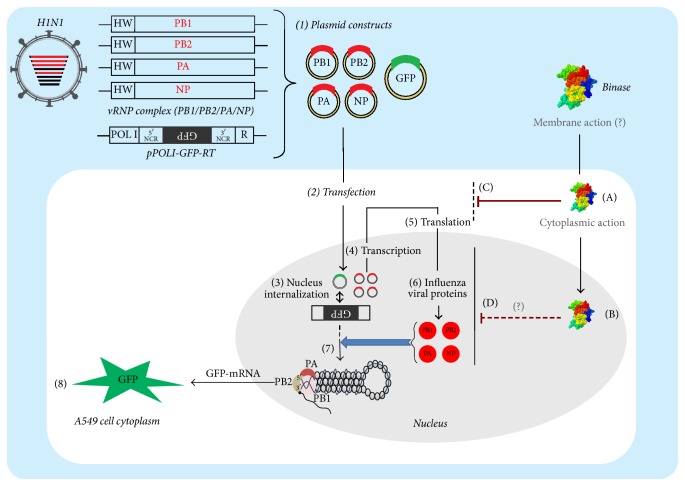
Hypothetic scheme of binase influence on functional viral RNP polymerase complex formation monitored by GFP fluorescence in the cells using the influenza A virus mini genome system. For the in vitro reconstitution of viral ribonucleoprotein complex (vRNP), plasmids pHW-PB1-Hamburg, pHW-PB2-Hamburg, pHW-PA-Hamburg, and pHW-NP-Hamburg were used. They carry genes for generating the mRNAs of polymerases and NP segments of H1N1pdm virus. These plasmids were transfected into A549 cells along with pPOLI-GFP-RT plasmid, which generates a vRNA-like POLI-transcript encoding the green fluorescent protein. GFP gene in a latter construct is encoded in negative-sense and flanked by the 3′- and 5′-noncoding region of the NS RNA segment of influenza A/WSN/33 virus [2] placed between a truncated human RNA polymerase I promotor (POLI) and hepatitis delta virus ribozyme (R). The expressed subunits of the viral polymerases and nucleoprotein replicate and transcribe the influenza virus-like RNA expressed by pPOLI-GFP-RT into GFP mRNA, resulting in the detection of GFP activity in transfected cells. The described steps are labeled as follows: (1) plasmid constructs encoding vRNP subunits and GFP as a reporter protein, (2-3) transfection and internalization of plasmids into nucleus of A549 cells, (4–6) expression of viral polymerase complex, (7) maturation of GFP transcript by viral polymerase complex, and (8) GFP signaling. Possible binase actions are represented in the steps: (A) binase internalization into cytoplasm, (B) binase internalization into nucleus, (C) and (D) inhibitory effect of binase on the expression of influenza A virus mini genome system in cytoplasm and nucleus, respectively.

In Figure 4, we have shown a scheme reflecting different possible mechanisms of a binase effect on influenza A viral functional ribonucleoprotein (RNP) complex gene expression using a vRNA-like Pol1-transcript encoding a reporter gene (GFP) step by step starting from internalization of binase (steps A and B) to the step of blocking of viral translation (steps C, D). Steps depicting production of reporter mRNA (7-8) and transcription of viral RNP mRNA (4-5) can be inhibited by binase.

**Figure 5:**
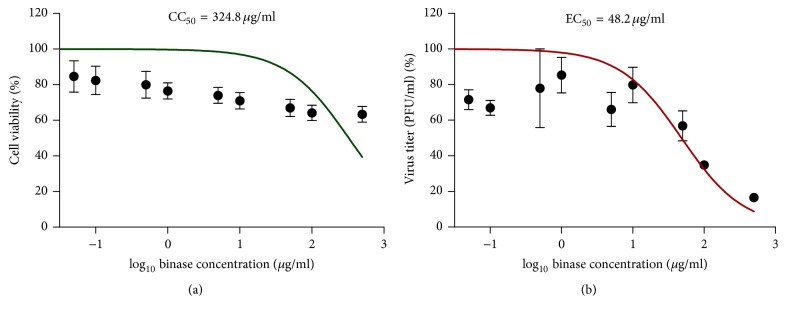
Viability of HeLa cells treated by binase (24 h) (a) and the influence of binase on HRV1A virus titer after 24 h incubation of HeLa cells with different concentrations of the RNase. CC_50_ and EC_50_ are the binase concentration, which induces 50% cell death and 50% reduction of virus titer, respectively. 1 *μ*g/*μ*L of binase corresponds to 82 *μ*M. The* R*-squared value and cytotoxic and effective concentrations were calculated using GraphPad Prism 5.0 Software.

Based on the conducted experiments, we can conclude that binase acts on synthesized positive-sense mRNA; however, we do not rule out that binase would digest negative-sense vRNA of H1N1pdm virus or its cRNA. With respect to vRNA it seems less possible, because vRNA is protected by NP proteins and, moreover, its degradation would result in complete elimination of the virus from the cell culture. To confirm the ability of binase to degrade positive-sense RNA of virus, which ignores the internalization and localization in nuclei in order to serve as a template during translation of viral proteins, we used a different system: HeLa cells were infected by positive-sense RNA-containing HRV1A virus. In HeLa cells HRV1A virus is easily propagated; it replicates in HeLa cells to high titers. We show binase is nontoxic for HeLa cells in concentrations under 100 *μ*g/ml (Figure 5(a)). It reduced the titer of HRV1A virus by 3 times at 100 *μ*g/ml (Figure 5(b)). The results confirmed the possibility of binase to degrade viral positive-sense RNA without internalization into the nucleus. Thus, regardless of the negative- or positive-sense genome of single stranded RNA viruses, binase might be able to cleave viral RNA catalytically and thereby inhibit the synthesis of the viral proteins. This property of the bacterial enzyme uncovers the promising aspects of its application as a new antiviral agent.

To replicate successfully, viruses protect their genomic material from degradation by the host cell. RNA viruses must contend with numerous destabilizing host cell processes including mRNA decay pathways and viral RNA degradation resulting from the antiviral response [18]. However, viruses do not possess specific defense system against bacterial RNases applied exogenously, and viral mRNA remains an important target for antiviral drug discovery.

To impair viral RNA transcription, different strategies have been used. For example, siRNA duplexes directed against the Rift Valley fever virus (RVFV) nucleoprotein can effectively inhibit RVFV replication in human (MRC5 cells) and African green monkey cells (Vero E6 cells). It was shown that pretreatment of cells with the nucleoprotein-specific siRNAs markedly reduces the virus titer [19]. Protein-protein interactions of PB1-PB2 subunits play pivotal roles in assembling the functional polymerase complex, which is essential for the replication and transcription of influenza virus RNA. Novel small molecule compounds which inhibited the interaction of RNA-dependent RNA polymerase subunits were identified. Recent efforts of discovery of antiviral drugs targeting RNA have provided drug-like small molecules that inhibit viral replication and include inhibitors of human immunodeficiency virus (HIV), hepatitis C virus (HCV), severe acute respiratory syndrome coronavirus (SARS CoV), and influenza A virus [20]. Highly conserved noncoding RNA (ncRNA) elements in viral genomes and transcripts offer new opportunities to expand the repertoire of drug targets for the development of anti-infective therapy. Viruses for which targeting ncRNA components in the genome or transcripts may be promising include insect-borne flaviviruses (Dengue, Zika, and West Nile) and filoviruses (Ebola and Marburg) [21].

According to our data, both negative-sense and positive-sense RNA-containing viruses can be targeted by binase. The +ssRNA transcript of influenza virus is used as mRNA for the synthesis of structural proteins; +ssRNA of rhinovirus is used both as mRNA and as the packaged genome. The rhinovirus +ssRNA does not need to be transported into the nucleus; the influenza +ssRNA is also located in the cytoplasm. Cytoplasmic allocation of +ssRNAs facilitates their targeting by binase. We have shown previously that massive internalization of binase into nucleus had already started after 3 h of binase incubation with A549 cells [17]. Therefore, we can propose that binase targets −ssRNA too.

## 4. Conclusion

We found that binase at nontoxic concentrations to eukaryotic cells does not alter the cellular protein production or mRNA expression but affects viral RNA production. Binase exerts its antiviral effect both against positive- and negative-sense RNA viruses suggesting binase as a promising antiviral agent against different viruses.

## Abbreviations

RNase: Ribonuclease
RI: RNase inhibitor protein
ACTB: *β*-Actin
GAPDH: Glyceraldehyde-3-phosphate dehydrogenase
RNP: Ribonucleoprotein
GFP: Green fluorescent protein
MOI: Multiplicity of infection
FFU: Focus forming units
p.i.: Postinfection
IEF: Isoelectric focusing
+ssRNA: Positive-sense single stranded RNA
−ssRNA: Negative-sense single stranded RNA.
